# Quantum Calculations on a New CCSD(T) Machine-Learned Potential Energy Surface Reveal the Leaky Nature of Gas-Phase *Trans* and *Gauche* Ethanol Conformers

**DOI:** 10.1021/acs.jctc.2c00760

**Published:** 2022-08-11

**Authors:** Apurba Nandi, Riccardo Conte, Chen Qu, Paul L. Houston, Qi Yu, Joel M. Bowman

**Affiliations:** † Department of Chemistry and Cherry L. Emerson Center for Scientific Computation, Emory University, Atlanta, Georgia 30322, United States; ‡ Dipartimento di Chimica, Università Degli Studi di Milano, via Golgi 19, 20133 Milano, Italy; § Independent Researcher, Toronto 66777, Canada; ⊥ Department of Chemistry and Chemical Biology, Cornell University, Ithaca, New York 14853, United States; ¶ Department of Chemistry and Biochemistry, Georgia Institute of Technology, Atlanta, Georgia 30332, United States; # Department of Chemistry, Yale University, New Haven, Connecticut 06520, United States

## Abstract

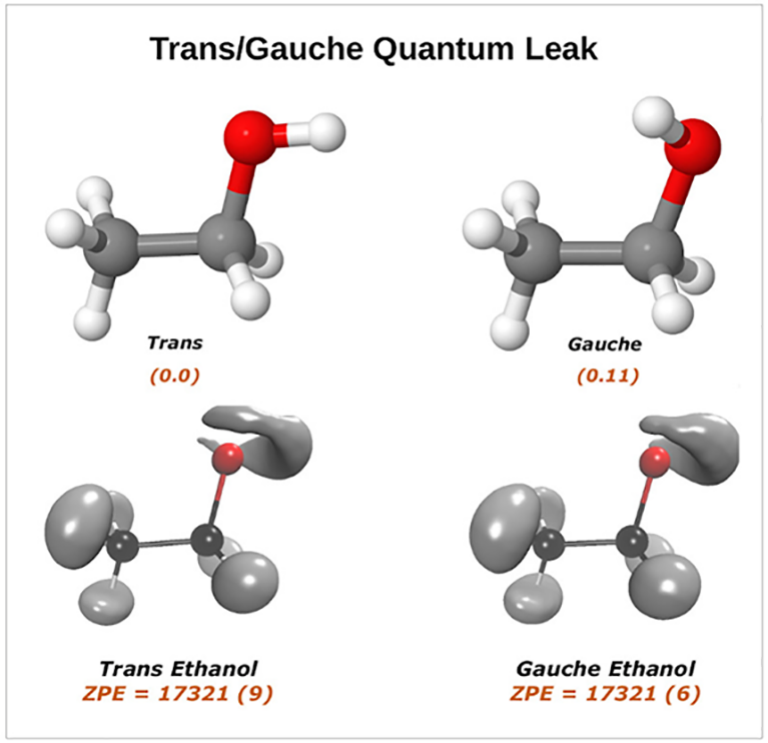

Ethanol is a molecule of fundamental interest in combustion, astrochemistry, and condensed phase as a solvent. It is characterized by two methyl rotors and *trans* (*anti*) and *gauche* conformers, which are known to be very close in energy. Here we show that based on rigorous quantum calculations of the vibrational zero-point state, using a new *ab initio* potential energy surface (PES), the ground state resembles the *trans* conformer, but substantial delocalization to the *gauche* conformer is present. This explains experimental issues about identification and isolation of the two conformers. This “leak” effect is partially quenched when deuterating the OH group, which further demonstrates the need for a quantum mechanical approach. Diffusion Monte Carlo and full-dimensional semiclassical dynamics calculations are employed. The new PES is obtained by means of a Δ-machine learning approach starting from a pre-existing low level density functional theory surface. This surface is brought to the CCSD(T) level of theory using a relatively small number of *ab initio* CCSD(T) energies. Agreement between the corrected PES and direct *ab initio* results for standard tests is excellent. One- and two-dimensional discrete variable representation calculations focusing on the *trans*–*gauche* torsional motion are also reported, in reasonable agreement with experiment.

## Introduction

Ethanol is one of the most important organic molecules with many applications in industrial products, chemicals, and solvents. It is also the leading biofuel in the transportation sector, where it is mainly used in a form of reformulated gasoline^1,2^ and studied from scientific, industrial, and environmental perspectives for its role in internal combustion engines.

Ethanol exists as a mixture of *trans* (or *anti*) and *gauche* (+/−) conformers in solid, liquid, and gaseous states.^3−5^ Therefore, the energy difference between the *trans* and *gauche* conformers is expected to be very small. This is corroborated by the data extracted upon fitting models to spectroscopic experiments in the microwave and far-infrared portion of the electromagnetic spectrum, which estimate the energy gap at 0.12 kcal/mol or 41 cm^–1^ in favor of the more stable *trans* conformer.^4,6^ Therefore, there is an anticipated preponderance of the *gauche* form at room temperature (62%) because of its 2-fold degeneracy (+/−). Furthermore, ethanol has two isomerization saddle points and a 3-fold methyl torsional potential, which make its potential surface much more complex.

Reports on ethanol in the literature have been often accompanied by several experimental studies of its isomers. In 1980, Quade and co-workers reported microwave torsional-rotational spectra of *gauche* ethanol,^4^ and later Durig and Larsen presented a detailed examination of the torsional modes.^6^ Rotational isomerization of ethanol in nitrogen and argon matrices has been recorded under various conditions of temperature and irradiation in the OH and CO stretches by Coussan et al.^7^ In 2013, a comparative analysis of low-temperature FTIR absorption spectra was reported for ethanol isolated in an argon matrix by Balevicius and co-workers.^8^ It was observed that in an argon matrix ethanol is predominantly in the *trans* configuration, although the most intense absorption lines of the *gauche* conformer were still observed in the spectra of the samples. Recently, the *trans*–*gauche* conformational distribution of ethanol has been investigated using the O–H and symmetric C–C–O stretching infrared spectra in argon and nitrogen matrix.^9^ It was found that the *trans* conformer is more populated in a nitrogen mixture, whereas the *gauche* conformer is more populated in the argon mixture. After thermal cyclization in the matrix, the *trans* conformer isomerizes to the *gauche* conformer in a nitrogen matrix, but the reverse happens in an argon matrix. Finally, Pearson et al. (PBD) also reported a comprehensive analysis of the 3-fold asymmetric rotational–torsional spectrum of ethanol in the torsional ground state of the OH internal rotation.^10^ Zheng et al. considered the partition functions of rotors in ethanol and performed helpful calculations on the energy levels.^11^

Ethanol has also been investigated extensively using electronic structure calculations to understand its energetics and complex potential energy surface (PES). In 2004, calculations have been performed at the MP2/aug-cc-pVTZ and CCSD(T)/aug-cc-pVTZ levels of theory by Dyczmons.^12^ It is reported that the *trans* isomer is 0.52 kJ mol^–1^ or 44 cm^–1^ more stable than the *gauche* isomer, and the energy barrier for the torsional motion of the OH group for *trans* to *gauche* isomerization is 3.9 kJ mol^–1^ or 326 cm^–1^. Recently, a high level calculation has been performed at the CCSD(T)/aug-cc-pVQZ level of theory by Kirschner and co-workers.^13^ It was found that the *trans* isomer is more stable by 0.53 kJ mol^–1^ or 44 cm^–1^ compared to the *gauche* isomer. Thus, it is concluded that the *trans* conformer is more stable in the gas phase compared to the *gauche* conformer. Remarkably, a thorough investigation on conformational analysis by systematically improving the basis set and the level of electron correlation of ethanol was reported by Kahn and Bruice in 2005.^14^ Their best estimate of the *trans*–*gauche* energy gap is 0.134 kcal mol^–1^ or 47 cm^–1^ and the energies of the two isomerization TSs (*eclipsed* and *syn*) are 1.08 kcal mol^–1^ or 378 cm^–1^ and 1.20 kcal mol^–1^ or 420 cm^–1^, respectively, relative to the *trans* minimum. They came to the common conclusion that the *trans* conformer is more stable in the gas phase compared to the *gauche* conformer. Very recently, Grimme and co-workers reported combined implicit and explicit solvation protocols for the quantum simulation of ethanol conformers in the gas phase, liquid phase, and in CCl_4_ solutions. The implicit treatment of solvation effects suggested that the ratio of the *trans* and *gauche* conformers of ethanol increases only slightly when going from gas phase to a CCl_4_ solution, and to neat liquid.^15^

However, we note that both experiments and theoretical calculations may not have been conclusive in describing the *trans*–*gauche* dichotomy of gas-phase ethanol. On the one hand, the experiments referenced above appear to deal with a mixture of the two conformers and to be even affected by experimental conditions. For instance, in ref (7), it is shown that infrared experiments performed in the 8–30 K temperature range point at temperature-dependent *trans*–*gauche* isomerism when a nitrogen matrix is employed, while the temperature dependence vanishes and evidence of *trans* isomer only is found when an argon matrix is used. Other experiments found a mixture of the two conformers also in argon matrix but with abundance conclusions at odds and an interconversion rate dependent on temperature and matrix type. On the other hand, accurate but static theoretical calculations have been performed only at the level of electronic structure, while quantum nuclear effects have not been taken into consideration or have been estimated just with basic and inaccurate harmonic approaches.

The main goal of this study is to investigate the energetics of ethanol and its challenging conformational properties including quantum nuclear effects. This is obtained by means of rigorous diffusion Monte Carlo (DMC) and semiclassical calculations able to describe nuclear quantum effects performed on a new “gold standard” *ab initio* CCSD(T) PES, which we have constructed for this investigation.

Developing high-dimensional, *ab initio*-based PESs remains an active area of theoretical and computational research. Significant progress has been made in the development of machine learning (ML) approaches to generate PESs for systems with more than five atoms based on fitting thousands of CCSD(T) energies.^16−19^ Examples of potentials for 6- and 7-atom chemical reactions which are fits to tens of thousands or even hundred thousand CCSD(T) energies have been reported.^20,21^ However, there is a bottleneck for developing the PES at high level theory with the increase of molecular size. Due to the steep scaling of the “gold standard” CCSD(T) theory (∼*N*^7^, *N* being the number of basis functions), it is computationally demanding to fit PESs for systems with a larger and larger number of atoms.

The increasing dimensionality of the PES with the increase of number of atoms requires a large number of training data sets to fit the PES. Thus, the use of lower-level methods such as density functional theory (DFT) and second-order Møller–Plesset perturbation (MP2) theory is understandable but probably not accurate enough for precise investigations like the one targeted here. To circumvent this bottleneck, researchers are applying ML approaches to bring a PES based on a low-level of electronic structure theory (DFT or MP2) to a higher level (CCSD(T)) one. One way to achieve this is by means of the Δ-machine learning (Δ-ML) approach, in which a correction is made to a property data set obtained using an efficient, low-level *ab initio* theory such as DFT or MP2.^22−27^

We apply a Δ-ML approach that we recently reported^27,28^ to take a DFT-level PES of ethanol that we recently reported^29^ (details of the data set of energies and gradients are given in that paper) to the CCSD(T) level using a manageable subset of *ab initio* CCSD(T) energy points. The new PES is tested against the usual fidelity tests and then employed for the challenging DMC and SC simulations and for an investigation of the wave functions of the −CH_3_ and −OH motions, for which Quade et al. have suggested a geared motion by analyzing microwave spectra.^30,31^

The paper is organized as follows. In the next section, we briefly summarize the theory of the Δ-ML approach for PES construction, and diffusion Monte Carlo and adiabatically switched semiclassical initial value representation for zero-point energy calculations. Then we present results with a discussion followed by a summary and conclusions.

## Theory and Computational Details

### Δ-Machine Learning for PES Construction

The theory underneath our Δ-ML approach is very simple^27,28^ and can be presented in a simple equation:
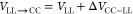
1where *V*_LL→CC_ is the corrected PES, *V*_LL_ is a PES fit to low-level DFT electronic data, and Δ*V*_CC-LL_ is the correction PES based on high-level coupled cluster energies. It is noted that the difference between CCSD(T) and DFT energies, Δ*V*_CC-LL_, is not as strongly varying as *V*_LL_ with respect to the nuclear configurations, and therefore, just a small number of high-level electronic energies is adequate to fit the correction PES. In the present application to ethanol, we computed a total of 2319 CCSD(T)-F12a/aug-cc-pVDZ electronic energies and performed training on a subset of these data in size of 2069 energies. This choice of basis was made to balance between accuracy and computational efficiency. We do compare several key energies at CCSD(T)-F12a/aVDZ and CCSD(T)-F12a/aVQZ level with the published results^13^ using CCSD(T)/aVQZ level of theory via single-point calculations (at the four stationary points). It turns out that the CCSD(T)-F12a/aVDZ gives virtually the same energetics as CCSD(T)-F12a/aVQZ (within few wave numbers). A table comparing the single-point energies at different basis is given in the Supporting Information (SI) (Table S1).

Here we employ permutationally invariant polynomial (PIP) approach to fit both the *V*_LL_ and Δ*V*_CC-LL_ PESs. The theory of permutationally invariant polynomial is well established and has been presented in several review articles.^16,17,32−34^ In terms of a PIP basis, the potential energy, *V*, can be written in compact form as
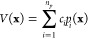
2where *c*_*i*_ indicates linear coefficients, *p*_*i*_ indicates PIPs, *n*_p_ is the total number of polynomials for a given maximum polynomial order, and ***x*** indicates Morse variables. For example, *x*_*αβ*_ is given by exp(−*r*_*αβ*_/λ), where *r*_*αβ*_ is the internuclear distance between atoms α and β. The range (hyper)parameter, λ, was chosen to be 2 bohr. The linear coefficients are obtained using standard least-squares methods for a large data sets of electronic energies (and for large molecules’ gradients as well) at scattered geometries.

In order to develop a corrected PES, we need to generate a data set of high and low-level energies for training and testing. In this study, we need both DFT and CCSD(T) data sets. Training is done for the correction PES Δ*V*_CC-LL_, and testing is done for the corrected PES *V*_LL→CC_. Do note that this two-step “training and testing” is on different data sets.

**Figure 1:**
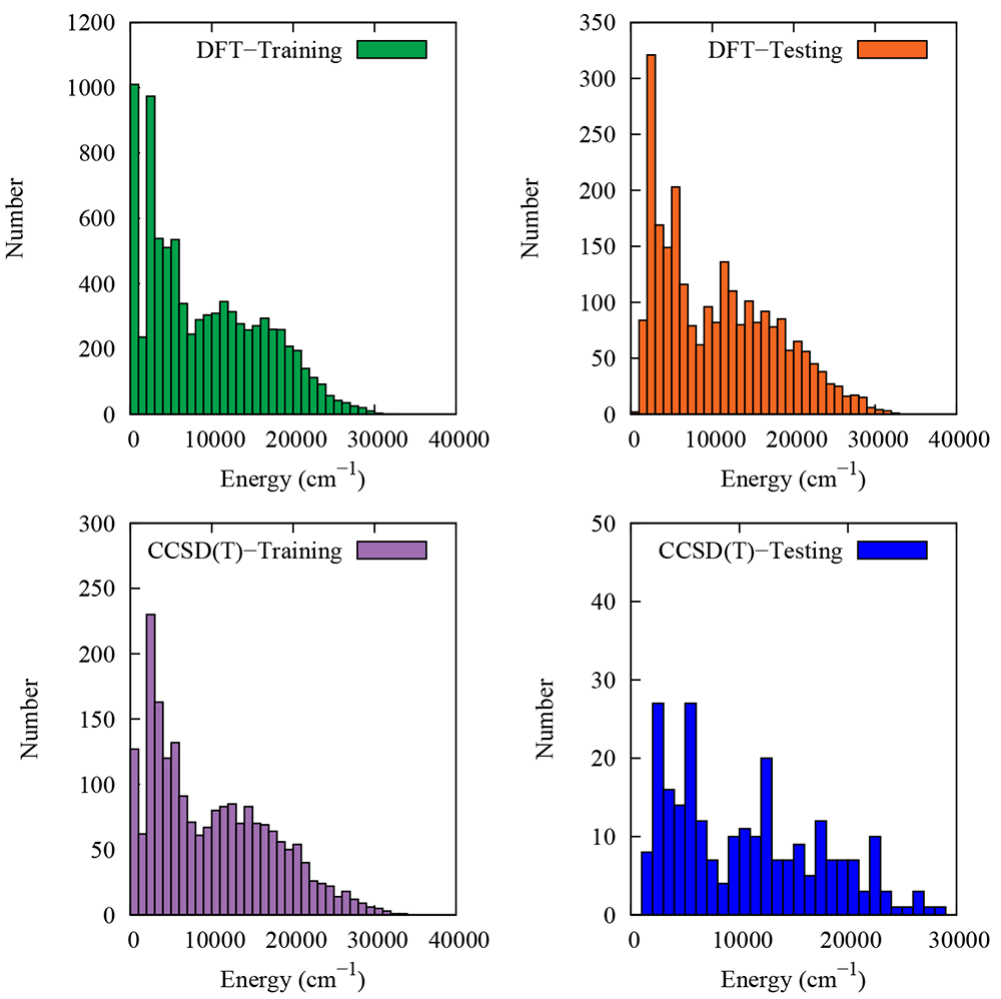
Distributions of DFT and CCSD(T) electronic energies (cm^–1^) of both training and test data sets relative to their respective minimum value.

Here we take the DFT data set from our recently reported “MDQM21” data set^29^ where a total of 11 000 energies and their corresponding gradients were generated from *ab initio* molecular dynamics (AIMD) simulations at the B3LYP/6-311+G(d,p) level of theory. The DFT PES (*V*_LL_) was a fit using 8500 DFT data points, which span the energy range of 0–35 000 cm^–1^. Here, we generate a sparse data set that contains CCSD(T)-F12a/aVDZ energies at 2319 configurations, taken from the “MDQM21” data set. The following procedure is employed to generate the data set of 2319 configurations. First, we took every eighth geometry from the DFT training data set of 8500 configurations, which gave a set of 1063 geometries. Then we took half of the DFT test data set of 2500 geometries. From the other half of the DFT test data set, we took just 6 geometries having energy greater than 30 000 cm^–1^ relative to the minima. These led to a total of 2319 configurations subject to CCSD(T) single point energy computation. This 2319-geometry data set is partitioned into a training data set of 2069 geometries and a test data set of 250 geometries, respectively. Histogram plots of the distribution of DFT and CCSD(T) electronic energies are shown in Figure 1, where it can be seen that both the DFT and CCSD(T) data sets span a similar energy range. Geometry optimization and normal-mode analysis are performed to examine the fidelity of the *V*_LL→CC_ PES.

### Diffusion Monte Carlo

This PES is also applied to compute rigorous quantum zero-point energies (ZPEs) of ethanol and its single deuterated isotopologues using unbiased DMC calculations. The concept behind DMC is to solve the time-dependent Schrödinger equation in imaginary time.^35−37^ This is done by simulating a random walk of many replicas, also called “walkers”, of the molecule, using a birth/death processes. At each step, a random displacement in each degree of freedom is assigned to each walker, and this walker may remain alive (and may give birth to a new walker) or be killed by comparing its potential energy, *E*_*i*_, with a reference energy, *E*_*r*_. For the ground state, the probability of birth or death is given as
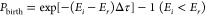
3
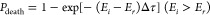
4where Δτ is the step size in imaginary time. After removing all dead walkers, the reference energy is updated using the equation
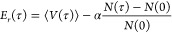
5where τ is the imaginary time, ⟨*V*(τ)⟩ is the average potential over all the walkers that are alive, *N*(τ) is the number of live walkers at time τ, and α is a parameter that can control the fluctuations in the number of walkers and the reference energy. Finally, the average of the reference energy over the imaginary time gives an estimate of ZPE.

In this study, each DMC trajectory is propagated for 30 000 time steps with step size of 5.0 au; 20 000 steps are used to equilibrate the walkers, and the reference energies in the remaining 10 000 steps are used to compute the ZPE. For each isomer, 15 DMC simulations (or trajectories) were carried out, and the final ZPE is the average of the 15 simulations. Statistical uncertainty of the zero-point energy is defined as the standard deviation of DMC energies over the total number of simulations. This is written as
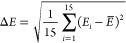
6where *E̅* is the average energy over the 15 simulations. We also perform DMC calculations on three single deuterated isotopologues employing 15 DMC trajectories. For *trans*- and *gauche*-CH_3_CH_2_OH and *trans*- and *gauche*-CH_3_CH_2_OD, 40 000 random walkers are used, while for CH_2_DCH_2_OH and CH_3_CHDOH, only 20 000 random walkers are employed.

We note that we have used DMC calculations of zero-point energies in numerous similar applications using ML potential energy surfaces. Some recent examples and additional details of our implementation can be found in refs (28), (38), and (39).

### Adiabatically Switched Semiclassical Initial Value Representation

Calculation of ethanol (*trans* and *gauche*) ZPEs and those of its deuterated isotopologues can be performed from a dynamical point of view by means of the adiabatically switched semiclassical initial value representation (AS SCIVR) technique. The goal is to corroborate DMC findings employing a completely different, but still full-dimensional, technique.^40−42^ AS SCIVR is a recently developed two-step semiclassical approach able to regain quantum effects starting from classical trajectories. In this it is quite similar to standard semiclassical techniques,^43−46^ but it differs in the way the starting conditions of the semiclassical dynamics run are selected. In AS SCIVR, preliminary adiabatic switching dynamics is performed. On the basis of the adiabatic theorem, this allows researchers to start from harmonic quantization and approximately preserve quantization after switching on the true system Hamiltonian. The exit molecular geometry and momenta of the adiabatic switching run serve as starting conditions for the subsequent semiclassical dynamics trajectory. This entire procedure is applied to a distribution of harmonically quantized starting conditions.

In practice, the adiabatic switching Hamiltonian is^47−49^
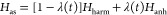
7where λ(*t*) is the following switching function
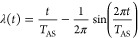
8*H*_harm_ is the harmonic Hamiltonian built from the harmonic frequency of vibration calculated by Hessian matrix diagonalization at the equilibrium geometry **q**_eq_, and *H*_anh_ is the actual molecular vibrational Hamiltonian. In our simulations, *T*_AS_ has been chosen equal to 25 000 au (about 0.6 ps) and time steps of 10 au have been employed. According to the Hamiltonian in eq 7, 5400 trajectories are evolved by means of a fourth order symplectic algorithm^50^ starting from harmonic ZPE quantization.

Once the adiabatic switching run is over, the trajectories are evolved according to *H*_anh_ for another 25 000 au with same step size to collect the dynamical data needed for the semiclassical calculation. This relies on Kaledin and Miller’s time-averaged version of semiclassical spectroscopy.^51,52^ Therefore, the working formula is
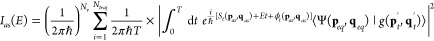
9where *I*_as_(*E*) indicates that a vibrational spectral density is calculated as a function of the vibrational energy *E*. *I*_as_ is peaked at the eigenvalues of the vibrational Hamiltonian, the lowest one being the ZPE. Eq 9 is made of several terms. *N*_v_ is the number of vibrational degrees of freedom of the system, that is, 21 in the case of ethanol. *T* is the total evolution time of the dynamics for the semiclassical part of the simulation. As anticipated, we chose *T* equal to 25 000 au with a time step size of 10 au.  is the instantaneous full-dimensional phase space trajectory. The semiclassical trajectory is started at time 0 from the final phase space condition (**p**_as_, **q**_as_) of the adiabatic switching part of the simulation. *S*_t_ is the classical action along the semiclassical trajectory, and ϕ_t_ is the phase of the Herman-Kluk pre-exponential factor based on the elements of the stability matrix and defined as
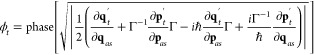
10where Γ is an *N*_v_ × *N*_v_ matrix usually chosen to be diagonal with elements numerically equal to the harmonic frequencies. We note that evolution in time of ϕ_t_ requires calculation of the Hessian matrix, which represents the bottleneck of the AS SCIVR approach and semiclassical methods broadly speaking. Based on Liouville’s theorem, the stability (or monodromy) matrix has the property to have its determinant equal to 1 along the entire trajectory. However, classical chaotic dynamics can lead to numerical inaccuracies in the propagation, so, following a common procedure in semiclassical calculations, we have rejected the trajectories based on a 1% tolerance threshold on the monodromy matrix determinant value. Finally, the working formula is completed by a quantum mechanical overlap between a quantum reference state |Ψ⟩ and a coherent state |*g*⟩ characterized by the following representation in configuration space:
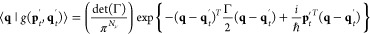
11The reference state |Ψ⟩ is usually chosen to be itself a coherent state. In eq 9, |Ψ⟩ is written as |Ψ(**p**_eq_, **q**_eq_)⟩, where **p**_eq_ stands for the linear momenta obtained in harmonic approximation setting the geometry at the equilibrium one (**q**_eq_).

AS SCIVR allows for a full-dimensional investigation of zero-point energies of ethanol isomers. It relies on high-energy classical molecular dynamics, and it is able to regain quantum effects by means of a stationary-phase approximation to Feynman’s quantum propagator. Therefore, AS SCIVR is a very different approach from the stochastic DMC one, and we employ it to corroborate the outcomes of DMC calculations. Numerous previous studies (the interested reader can have a look, for instance, at refs (53−55)) indicate that the method is able to approximate quantum results with an error ranging from very few wavenumbers to 20–30 cm^–1^. We expect to draw the same conclusions of the benchmark DMC calculation within this range of uncertainty. AS SCIVR can also provide information about excited states and quantum vibrational frequencies (including anharmonic overtones, combination bands, and Fermi resonances). Calculation of ethanol fundamental frequencies of vibration including Fermi resonances is left for a future work.

## Results and Discussion

### Starting Low Level PES (*V*_LL_)

The low level PES, *V*_LL_, was developed using the efficient B3LYP/6-311+G(d,p) level of theory. For the fit, we used maximum polynomial order of 4 with permutationally symmetry 321111, which led to a total of 14 752 PIPs in the fitting basis set. These were used to fit a data set of 8500 energies and their corresponding gradients. The fitting RMS errors for energies and gradients were 40 cm^–1^ and 73 cm^–1^ bohr^–1^, respectively. Testing was done on 2500 geometries. The testing RMS errors for energies and gradients were 51 cm^–1^ and 106 cm^–1^ bohr^–1^, respectively.

### Correction PES (Δ*V*_CC-LL_)

A data set of 2319 geometries was sparsely selected from the “MDQM21” DFT data set, and CCSD(T)-F12a/aug-cc-pVDZ energy computations were performed at those geometries. To develop the correction PES, we train Δ*V*_CC-LL_ on the difference between the CCSD(T) and DFT absolute energies of 2069 geometries and test the obtained surface on the remaining 250 geometries. A plot of Δ*V*_CC-LL_ versus the DFT energies is shown in the SI (in Figure S1) for both training and test data sets. Note that we reference Δ*V*_CC-LL_ to the minimum of the difference between the CCSD(T) and DFT energies (roughly 35 732 cm^–1^). As seen, the energy range of Δ*V*_CC-LL_ is about 1800 cm^–1^, which is much smaller than the DFT energy range relative to the minimum value (roughly 35 000 cm^–1^).

The difference Δ*V*_CC-LL_ is not as strongly varying as *V*_LL_ with respect to the nuclear configuration. Therefore, low-order polynomials will be adequate to fit the correction PES. We use maximum polynomial order of 2 with permutational symmetry 321111 to fit the training data set, which leads to a total of 208 unknown linear coefficients (equivalent to the number of terms in the PIP fitting basis set). These coefficients are determined by solving a linear least-squares problem. The PIP basis to fit this PES is generated using our “in-house” MSA software.^56,57^ The fitting RMS error of this Δ*V*_CC-LL_ fit is 25 cm^–1^. The fit is tested on the 250 energy differences and the RMS test error in this case is 41 cm^–1^.

### New CCSD(T) Ethanol PES (*V*_LL→CC_)

**Figure 2:**
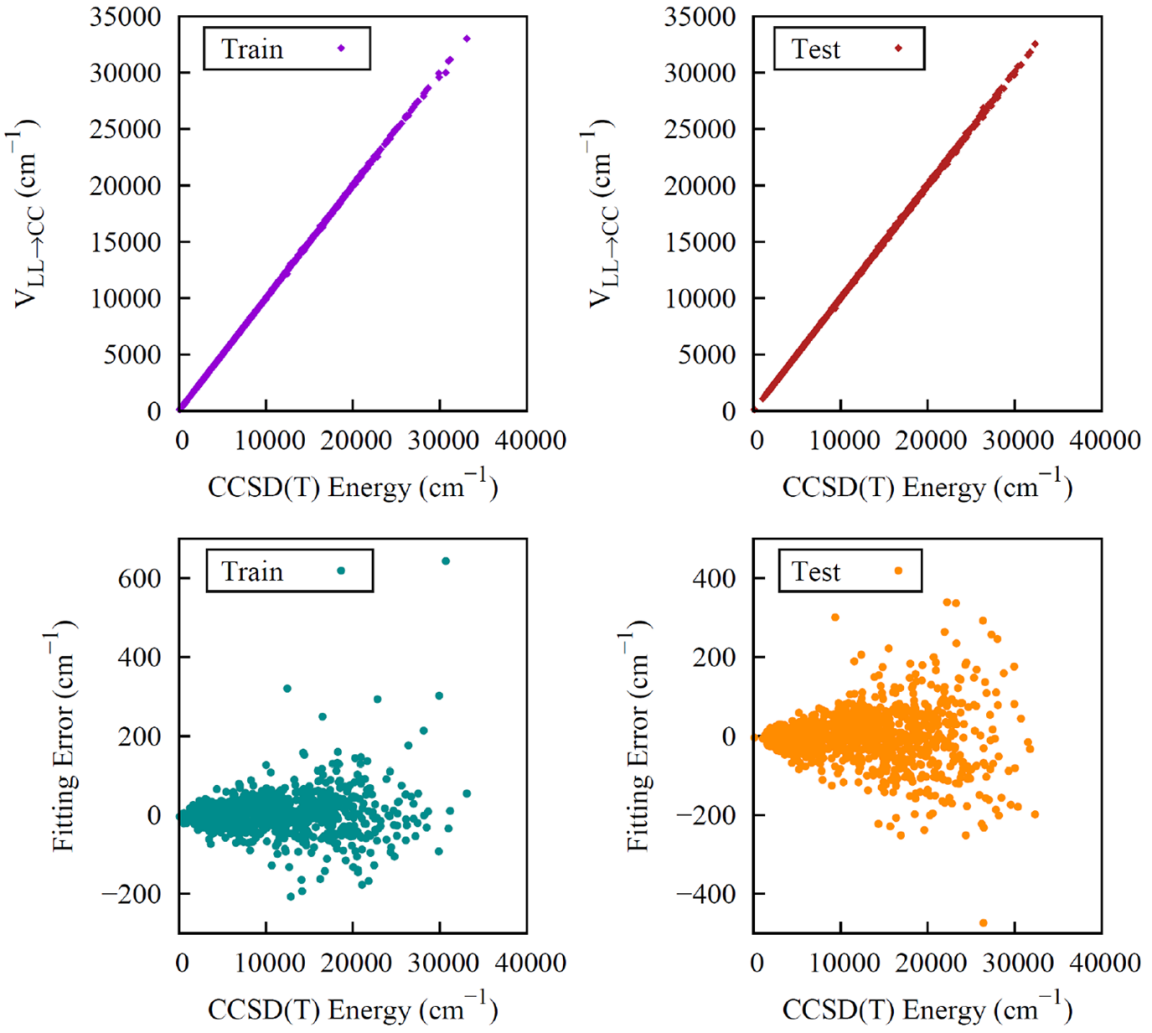
Two upper panels show energies of CH_3_CH_2_OH from *V*_LL→CC_ vs direct CCSD(T) ones for the indicated data sets. The one labeled “Train” corresponds to the configurations used in the training of Δ*V*_CC-LL_, and the one labeled “Test” is just the set of remaining configurations. Corresponding fitting errors relative to the minimum energy are given in the lower panels.

To obtain the CCSD(T) energies, we add the correction Δ*V*_CC-LL_ to the low-level DFT PES, *V*_LL_. A plot of *V*_LL→CC_ vs corresponding direct CCSD(T) energies for the training set of 2069 points and the test set of 250 points is shown in Figure 2. As seen, there is overall excellent precision; however, we see a few larger errors. The RMS differences between the *V*_LL→CC_ and direct CCSD(T) energies for the training and test data sets are 49 and 63 cm^–1^, respectively.

**Figure 3:**
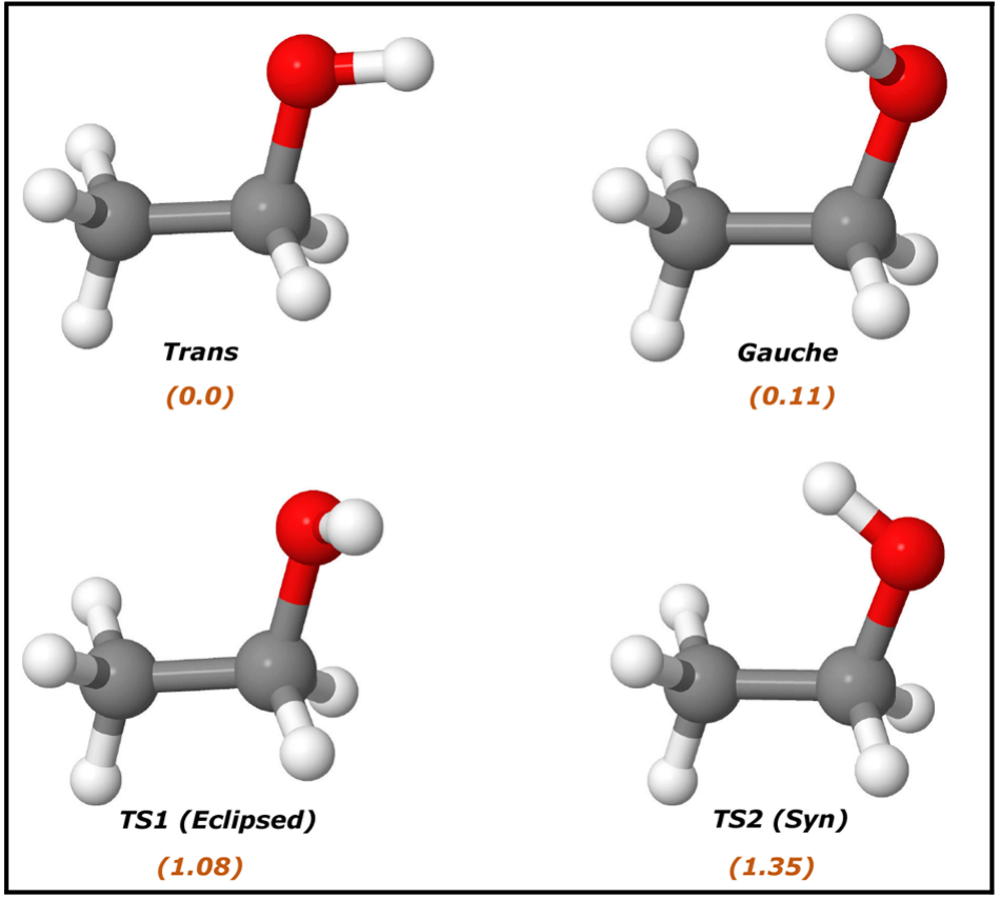
Geometry of *trans* and *gauche* conformers of ethanol, their two isomerization TSs, and their electronic energies (kcal/mol) relative to the *trans* minimum from Δ-ML PES.

To examine this fidelity of the new *V*_LL→CC_ PES, we perform geometry optimization and normal-mode frequency calculation of both *trans* and *gauche* isomers and their two isomerization saddle point geometries. They are the *eclipsed* one, in which the hydroxylic hydrogen eclipses with the hydrogen of the adjacent CH_2_ group, and the *syn* one, in which the hydroxylic hydrogen is above the methyl group. The structures of these isomers and saddle points are shown in Figure 3. We obtain the PES optimized energies within 5 cm^–1^ of the direct CCSD(T)-F12a calculation and find that the *trans* isomer is lower in energy by 38 cm^–1^. Next, to examine the vibrational frequency predictions of the PES, we perform normal-mode analyses for both *trans* and *gauche* isomers and their isomerization saddle points. The comparison of harmonic mode frequencies of *trans* and *gauche* ethanol with their corresponding *ab initio* ones is shown in Table 1. The agreement with the direct CCSD(T)-F12a/aug-cc-pVDZ frequencies is overall very good; the maximum error is 21 cm^–1^ for the lowest frequency mode of *trans* conformer, but most of the frequencies are within a few cm^–1^ of the *ab initio* ones, and the mean absolute error (MAE) is only 4 cm^–1^. The *gauche* isomer shows even better agreement with the *ab initio* data. The two *trans*–*gauche* isomerization saddle point geometries such as *eclipse* and *syn* ones are confirmed by obtaining one imaginary frequency. The normal-mode frequencies of this saddle point geometry are given in the SI (Table S2). The barrier heights of *trans*–*gauche* isomerization with respect to *eclipse* and *syn* TSs are found to be 377 and 472 cm^–1^, respectively, and the corresponding direct *ab initio* values are 389 and 438 cm^–1^. These are in excellent agreement with the experimental barrier heights of 402 and 444 cm^–1^.^6^ We also compare these CCSD(T) PES results with the DFT PES,^29^ and we note the fact that the DFT gives fortuitously excellent accuracy in this case. However, for the high frequency modes, we see a difference of 20 cm^–1^ with respect to CCSD(T) PES. More details are provided in Tables S4–S6 in the SI.

**Table 1:** Comparison of Harmonic Frequencies (in cm^–1^) between *V*_LL→CC_ PES and Corresponding *Ab Initio* (CCSD(T)-F12a/aug-cc-pVDZ) Ones of Both *Trans* and *Gauche* Isomers of Ethanol

	*trans*-thanol			*gauche*-ethanol		
mode	Δ-ML PES	*ab initio*	diff.	Δ-ML PES	*ab initio*	diff.
1	243	222	–21	268	258	–10
2	273	274	1	278	271	–7
3	417	413	–4	424	420	–4
4	818	813	–5	804	803	–1
5	909	907	–2	894	895	1
6	1055	1049	–6	1075	1069	–6
7	1115	1115	0	1094	1096	2
8	1181	1180	–1	1144	1141	–3
9	1284	1274	–10	1290	1284	–6
10	1302	1300	–2	1375	1374	–1
11	1403	1402	–1	1406	1402	–4
12	1454	1456	2	1424	1426	2
13	1488	1484	–4	1490	1491	1
14	1500	1501	1	1496	1497	1
15	1530	1531	1	1519	1522	3
16	2995	3001	6	3007	3014	7
17	3028	3036	8	3020	3028	8
18	3036	3042	6	3088	3089	1
19	3120	3122	2	3108	3108	0
20	3126	3127	1	3121	3123	2
21	3862	3853	–9	3845	3837	–8

**Figure 4:**
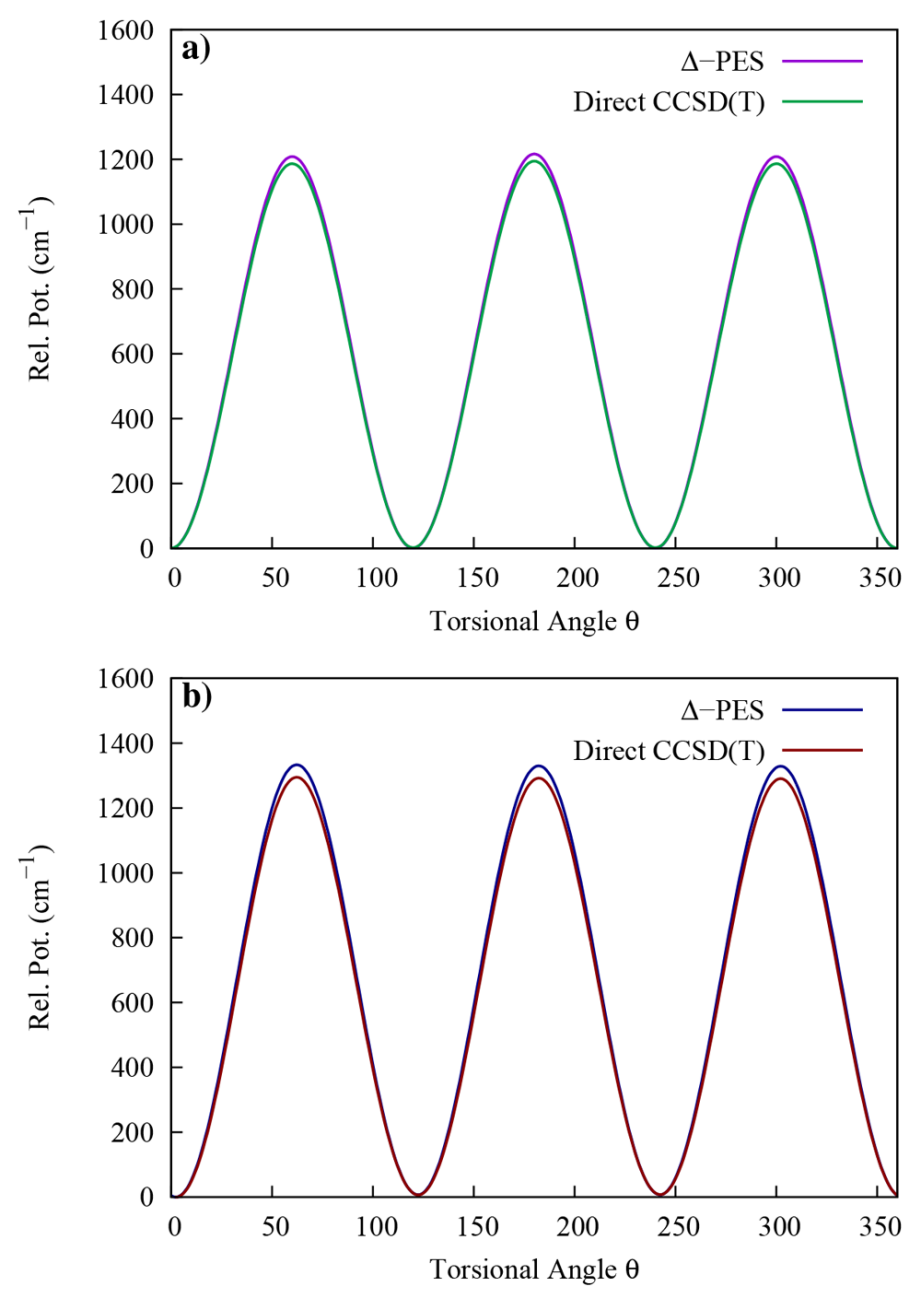
Comparison of torsional potential (not fully relaxed) of the methyl rotor of (a) *trans* and (b) *gauche* ethanol between direct CCSD(T) and Δ-ML PES.

Another comparison to the experiment we are able to perform thanks to the new PES concerns the torsional barrier for the methyl rotor. The methyl rotor torsional potentials (not fully relaxed) for both *trans* and *gauche* isomers as a function of the torsional angle are shown in Figure 4. It is seen that results from the PES are very close to the ones obtained from direct *ab initio* calculations at CCSD(T) level by means of a set of single point calculations. We obtain that the methyl torsional barriers for *trans* and *gauche* isomers are 1208 and 1324 cm^–1^, respectively. The methyl torsional barrier heights extrapolated from microwave spectroscopy for the *trans* and *gauche* isomers are 1174 and 1331 cm^–1^.^4,58,59^ A different experimental analysis of the infrared and Raman spectra determined the methyl torsional barriers to be 1185 and 1251 cm^–1^ for *trans* and *gauche*, respectively.^6^ To complete our investigation of torsional barriers, in the SI (Figure S2), we report the methyl rotor torsional potential (not fully relaxed) for TS1 and TS2 geometries as a function of the CH_3_ torsional angle. We get perfect 3-fold symmetry with barrier heights of 1283 and 1404 cm^–1^, respectively.

This is another proof of the accuracy of the new PES and another evidence of experimental results obtained from ethanol vibrational spectroscopy being inconclusive. So far only electronic energies have been investigated, but we now move to consider nuclear quantum effects.

As a remarkable quantum nuclear application of the PES, we present the results of diffusion Monte Carlo (DMC) calculations of the zero-point energy (ZPE) for both *trans* and *gauche* isomers and singly deuterated isotopologues. In addition to that, it is well-known that a DMC calculation is a very challenging test to examine the quality of a PES in extended regions of the configuration space. A common issue in PES fitting is the unphysical behavior in the extrapolated regions where the fitting data set is lacking data, and this is dramatically manifested by large negative values. These are referred to as “holes” in the PES. Generally, we have observed that “holes” occur for highly repulsive configurations, that is, short internuclear distances. Adding some more data in these regions and performing a refit generally eliminates the issue. Therefore, one goal of presenting DMC calculations is also to demonstrate that our PES correctly describes the high energy regions of ethanol and it is therefore suitable for quantum approaches that need to sample these regions.

Table 2 shows the DMC ZPEs of ethanol (both isomers) and singly deuterated isotopologues of the *trans* conformer along with semiclassical and harmonic ZPEs. It is seen that the agreement between AS SCIVR and DMC ZPEs is very good and within method uncertainties (for SC methods uncertainty is typically within 20–30 cm^–1^). Relative to the electronic global minimum, that is, the bottom of the *trans* conformer well, the DMC ZPEs of *trans* and *gauche* isomers are 17321 ± 9 cm^–1^ and 17321 ± 6 cm^–1^, respectively, whereas the corresponding SC ones are 17298 and 17317 cm^–1^, and the harmonic ZPEs are 17568 and 17621 cm^–1^. The harmonic ZPEs of the *trans* and *gauche* overestimate the true ZPE values by about 250–300 cm^–1^ revealing a substantial level of anharmonicity. We note that in the DMC calculations very few “holes” are detected and in just a couple of trajectories. The total number of “holes” detected is 44, which is negligible compared to the total number of configurations (of the order of 10^11^) sampled during the DMC trajectory calculations. This demonstrates that our PES can be in practice considered “hole-free”. A further certification of this is given by the AS SCIVR simulations, which are successfully run at energies close to the ZPE one. During DMC propagation, when a random walker encounters a “hole” (and thus it enters a region of large potential energy), we kill that walker and let the trajectory continue to propagate. This procedure follows our unbiased DMC algorithm.

**Table 2:** Harmonic, DMC, and SC ZPEs (cm^–1^) of *Trans* and *Gauche* Ethanol and Singly Deuterated Isotopologues^a^

molecule	harmonic ZPE	DMC ZPE	SC ZPE
CH_3_CH_2_OH (*trans*)	17568	17321 (9)	17298
CH_3_CH_2_OH (*gauche*)	17621	17321 (6)	17317
CH_3_CH_2_OD (*trans*)	16842	16619 (6)	16598
CH_3_CH_2_OD (*gauche*)	16894	16619 (8)	16611
CH_2_DCH_2_OH (*trans*)	16874	16649 (7)	16622
CH_3_CDHOH (*trans*)	16836	16613 (9)	16586

a Zero energy is set at the electronic global minimum. Values inside the parentheses represent statistical uncertainties in the DMC results.

We believe this is the first time the quantum anharmonic ZPE of ethanol is reported at CCSD(T) level of theory. At this point, a comparison of our values to the experimentally derived ones is very insightful. Since the experiment has the ZPE in it, we compare our DMC and SC results with 41 cm^–1^, which is the experimental energy difference value we already anticipated in the Introduction. The bare electronic energy difference on the PES is 38 cm^–1^ with the *trans* conformer being the lower energy one. SC calculations estimate an energy difference of 19 cm^–1^ still in favor of the *trans* conformer, while DMC results have the two conformers basically degenerate. These values suggest an energy gap narrower than the experimentally derived one, with SC and DMC results in agreement within uncertainty.

**Figure 5:**
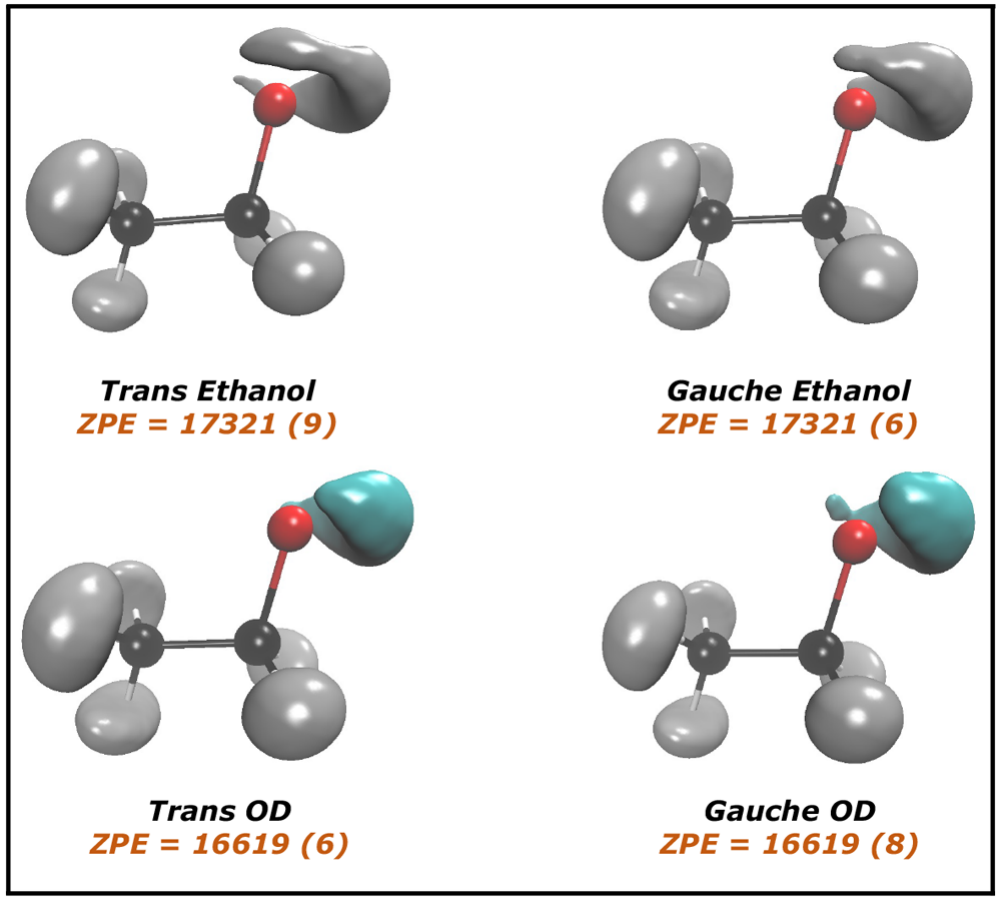
Vibrational ground-state wave function. The two upper panels represent the *trans*- and *gauche*-ethanol and the two lower panels represent the *trans*-CH_3_CH_2_OD and *gauche*-CH_3_CH_2_OD. The hydrogen atom attached to the oxygen atom has been removed to help the eye. ZPEs values are reported with uncertainties in parentheses.

**Figure 6:**
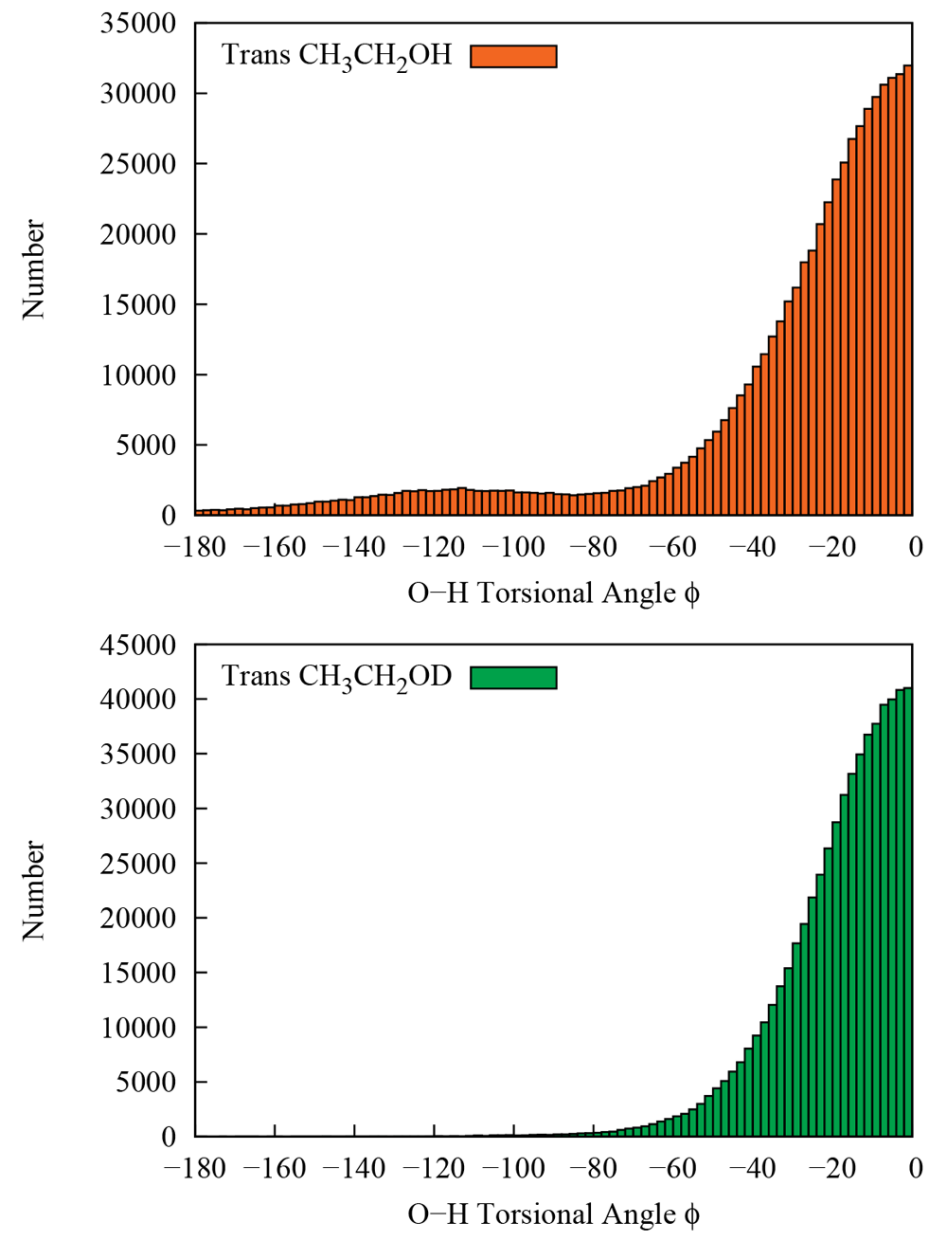
Distribution of C1–C2–O–H torsional angle (ϕ) from the DMC walkers. The upper panel represents the *trans*-CH_3_CH_2_OH and the lower panel represents the *trans*-CH_3_CH_2_OD.

The DMC vibrational ground-state wave functions for hydrogens for both *trans* and *gauche* conformers are shown in Figure 5. The DMC results clearly show that the ground-state wave function has a *trans* fingerprint even when starting from the *gauche* conformer. On the other hand, the ground-state wave function is partly delocalized at the *gauche* geometry. This conclusion is corroborated by the top panel of Figure 6, which shows the distribution of walkers at the end of DMC trajectories (15 DMC trajectories are computed, so total number of walkers are roughly 15 × 40  000 = 600 000) started from the *trans* configuration relative to the C1–C2–O–H torsional angle. The *gauche* geometry is found at the torsional angle of ±120 degrees.

We also present the vibrational ground-state wave function from DMC calculations for the OD motion in *trans*-CH_3_CH_2_OD, that is, one of the singly deuterated isotopologues of *trans* ethanol in Figure 5. The ZPEs for the deuterated isotopologue are still equivalent with very similar wave functions. In the case of deuteration, the bottom panel of Figure 6 shows that the torsional angle distribution is more centered at the *trans* geometry and only very few walkers are found at *gauche* geometry. This shows that, on one hand, quantum delocalization is somewhat quenched by the deuteration, while on the other hand, starting from the deuterated *gauche* conformer still leads to the deuterated *trans* one.

The wave function of the OD motion still looks partly delocalized, but an interesting effect of deuteration on the dynamics of ethanol can be pointed out by examining AS SCIVR calculations. In fact, as anticipated, a certain rate of AS SCIVR trajectories are numerically unstable and discarded according to a threshold parameter, as defined in the Theory and Computational Details section. The rejection rate we find is about 55% for both the *trans* and *gauche* conformers and also for the methyl-deuterated isotopologues. Conversely, for CH_3_CH_2_OD, the rejection rate decreases to about 20% and 38% for the *trans* and *gauche* conformer, respectively. This somehow strengthens DMC calculations by providing evidence of a more vibrationally localized motion for OD with respect to OH and a clue of a reduced influence of the “leak” effect.

**Figure 7:**
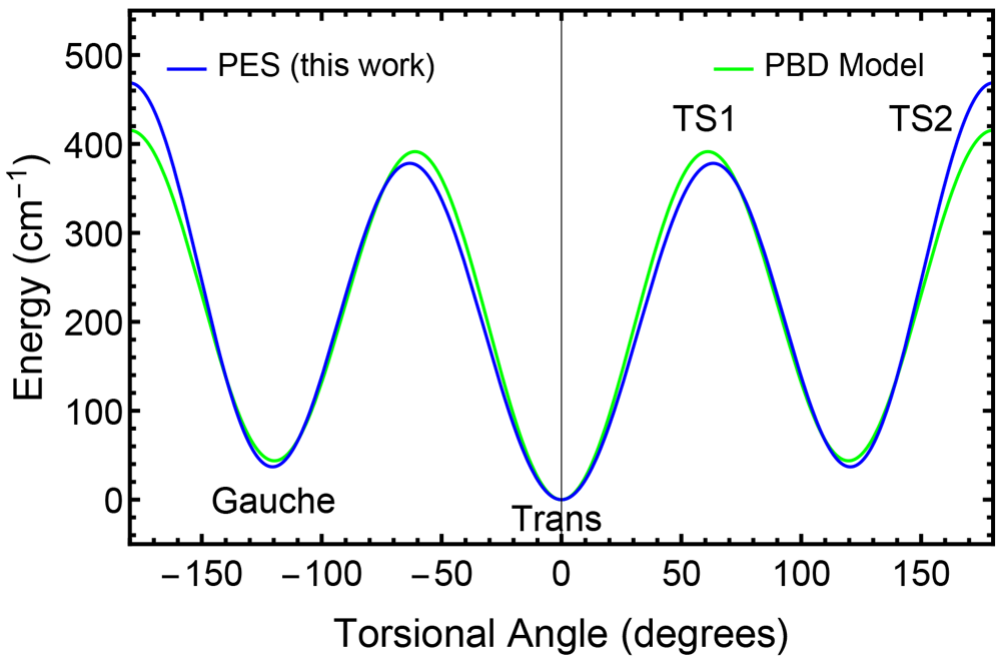
Comparison of C1–C2–O–H torsional potentials from this work (blue) and from PBD^10^ (green).

Then it is interesting to compare the 1-D O–H torsional potential determined from our full-dimensional PES with the model used by Pearson, Brauer, and Drouin (PBD).^10^ As shown in Figure 7, the two are very similar. The relative potential energies of the *gauche* state and TS1 with respect to the *trans* state are nearly the same, while TS2 is somewhat higher in energy for the 1-D potential from our PES as compared to PBD. Recall that the 1-D OH torsional is not fully relaxed, so some minor adjustments to it might be anticipated.

**Figure 8:**
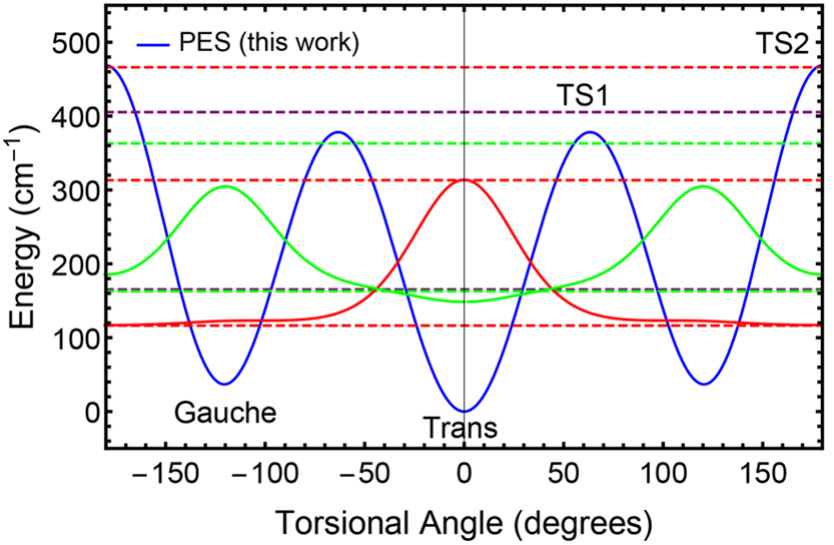
DVR results for energies and wave functions based on the 1-D C1–C2–O–H torsional potential from this work. The solid blue curve gives the potential, while the dotted lines give the first seven energy levels (there are two levels at 163.1 and 165.3 cm^–1^). The solid red and green lines give the wave functions corresponding to the two lowest torsional energy levels.

Having a 1-D model available is always advantageous because one can easily compute the energy levels and the corresponding wave functions. Our preferred method for doing so is by using the discrete variable representation (DVR) techniques described in ref (60). For the problem at hand, we use the azimuthal (0 to 2π interval, periodic) variant. There is really only one adjustable parameter, the moment of inertia of the rotor. An estimate for this might be μ_*O*–*H*_ × *r*_*OH*_^2^, where μ_O–H_ is the reduced mass of the OH in atomic units, and *r*_OH_ is the equilibrium distance of the O–H bond in bohr. For ethanol, this is about 3.2/(*N*_AV_*m_e_), where *N*_AV_ is Avogadro’s number and *m*_e_ is the mass of the electron. We reduced this numerical value from 3.2 to 2.7 so that when applied to the PBD model torsional potential, we obtained agreement with their energy differences. With this moment of inertia then applied to our own PES, we obtained the energy levels and wave functions shown in Figure 8, where the wave functions for only the first two levels are shown. It is interesting to note that there is substantial wave function amplitude for the *gauche* state at the geometry of the *trans* state and for the *trans* state at the geometry of the *gauche* state, an observation that was shown for the *trans* state also in the DMC results of Figure 6 based on the full-dimensional PES. In fact, the DMC *trans* wave function from Figure 6 and the wave function from Figure 8 are nearly identical, as shown in the SI in Figure S5.

**Figure 9:**
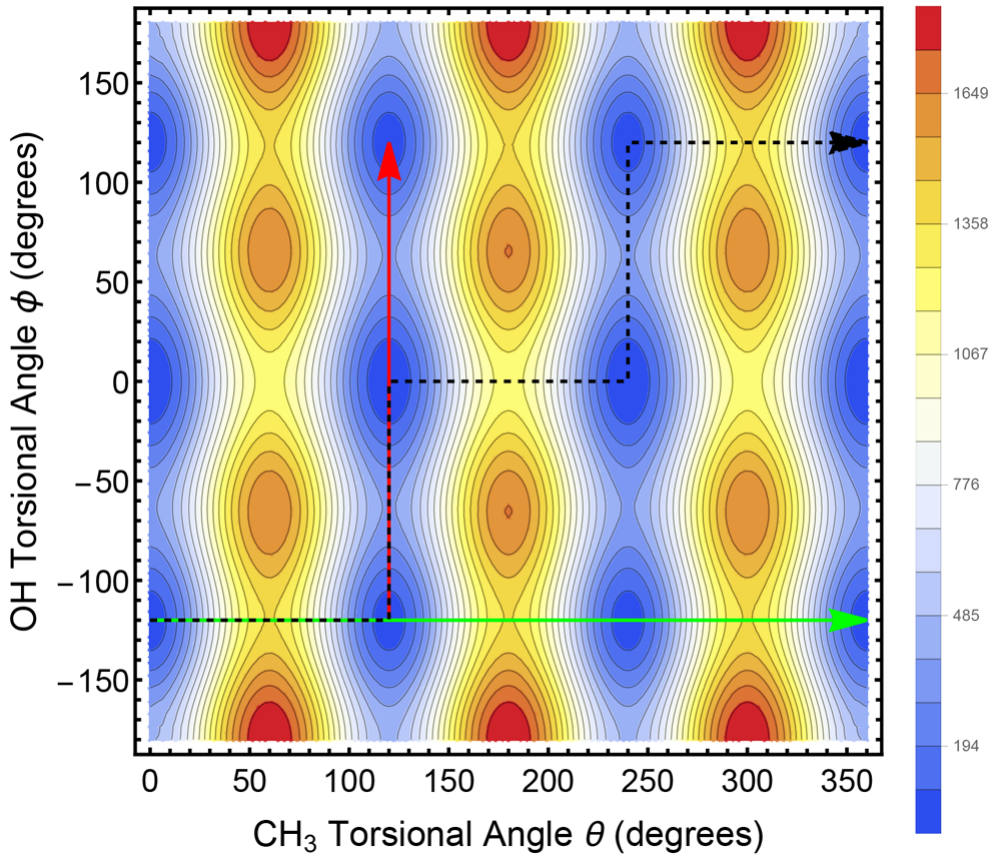
Two-dimensional contour plot of the potential energy as a function of OH torsional angle and CH_3_ torsional angle. The color scale gives the potential in cm^–1^. If the CH_3_ torsional angle is constant, for example, at 0°, 120°, 240°, or 360°, the lowest energy path for the OH rotational motion is in the vertical direction, for example, along the red arrow. If the OH torsional angle is constant, for example, at −120°, 0°, or 120°, then the lowest energy path for CH_3_ rotational motion is in the horizontal direction, for example, along the green arrow. If both the OH and CH_3_ are rotating, instead of moving in a straight line, say from {θ, ϕ} = {0°, −120°} to {360°, 120°}, the lowest energy path is to move along the sawtooth arrow, which describes a geared motion in which the horizontal and vertical displacements take place along the minimum energy paths.

Of course, a 1-D potential tells only a small part of the story. Two cuts of the 1-D CH_3_ torsional potential have previously been shown in Figure 4. When we combine these cuts with two others (taken at the OH torsional angles corresponding to TS1 and TS2, see Figure S2 in the SI) as well as with the OH torsional potential of Figures 7 and 8, we can obtain a reasonable fit for a 2-D potential of the combined motions of the OH and the CH_3_, as shown in Figure 9. As described in the caption, when both OH and CH_3_ are rotating, the minimum energy path for moving, for example, from the well at {θ, ϕ} = {0°, −120°} to {360°, 120°} is not at all straight but rather follows the dashed black sawtooth path reflecting the geared motion of the two rotors. As anticipated in the Introduction, this geared motion in ethanol has been suggested previously by Quade and colleagues from analysis of microwave spectra, but, to our knowledge, it has not previously been shown via a full-dimensional PES. The functional form we used to fit these potential cuts and then used for Figure 9 is given in the SI, along with Figure S3 showing the DVR results for the CH_3_ torsional potential, and Figure S4, which shows the OH torsional potential for θ = 0 and θ = 60 degrees.

The functional form of the 2-D torsional motions just mentioned also made it possible to perform a 2-D DVR calculation of the combined energy levels and wave functions. Moments of inertia of 2.7/(*M*_AV_*m*_e_) for the OH rotor and 10.5/(*M*_AV_*m*_e_) for the CH_3_ rotor gave the best agreement with the experimental data summarized in PBD.^10^ The results are shown in Table 3, where the first column gives the observed transitions, the second column gives our transition estimates based on the 2-D model (which was fit to five cuts through the full dimensional PES, see SI), and the third column gives the DVR results if instead of the full model potential, we use a separable potential having no cross terms between functions of θ and ϕ. The agreement is good, though certainly not perfect. It should be noted, however, that the 2-D potential is based on unrelaxed cuts and on a fit to 5 cuts of the potential; other cuts could modify the 2-D fit to the full-dimensional surface. There could be adjustments due to either effect. Nonetheless, it is remarkable that the *ab initio* surface is in such reasonable agreement with the experiment. Following our calculations, we found that Zheng et al. had recommended moments of inertia for the two rotors based on their electronic structure calculations and the resulting low-lying energy levels. Their results, converted to atomic units, are 2.66/(*M*_AV_*m*_e_) for the OH rotor and 9.32/(*M*_AV_*m*_e_) for the methyl rotor, very close to the values we found to be in best agreement with the experimental results of PBD.

**Table 3:** Comparison of Experimental Energy Levels^10^ Relative to the Lowest Level, Our 2-D DVR Calculations, and Our 2-D DVR Calculations Omitting Cross Terms in the 2D Torsional Potential^a^

level	v_*OH*_	v_*CH3*_	experiment	full 2-D potential	omitting cross terms
e_1_	0	0	0	0	0
e_1_	0	0	39.5	52.3	46.7
o_1_	0	0	42.8	54.4	48.9
o_2_	1	0	202.6	198.2	196.9
e_2_	1	0	238.6	236.	235.1
o_3_	1	0	285.9	293.6	289.
e_0_	0	1	244.4	251.8	246.5
e_1_	0	1	?	299.4(?)	281.9(?)
o_1_	0	1	?	301.4(?)	284.9(?)
e_0_	0	2	475.5	472.1477.7	468.473.
e_1_	0	2	529.49	532.	504.4
o_1_	0	2	532.8	533.8	524.4

a All energies are in cm^–1^.

## Summary and Conclusions

We presented a new potential energy surface for ethanol at the CCSD(T) level of theory. This was achieved by a Δ-ML method applied to a recent B3LYP-based PES that we previously reported. The new PES was validated for torsional barriers and harmonic frequencies against direct CCSD(T) calculations for the *trans* and *gauche* conformers and their isomerization TSs. Diffusion Monte Carlo and semiclassical calculations were reported for the zero-point energies of CH_3_CH_2_OH and several singly deuterated isotopologues. DMC wave functions have also been presented.

Our main goal was to investigate the energetics of ethanol, which was known to be characterized by two conformers very close in energy. To achieve this goal, we needed a way to perform high-level quantum stochastic and dynamical simulations. Therefore, our first effort was to construct a “gold-standard” PES of ethanol suitable for quantum calculations that require sampling of the high energy region of the phase space. This was a real need for accurate quantum simulations and not just an exotic requirement. The DMC and AS SCIVR applications reported demonstrate that we not only achieved our goal but also that the PES is robust for application of methods spanning a large portion of the configuration space.

Our quantum results provided us with a breakthrough in the chemistry of ethanol since we found that the ground state is of *trans* type with a leak to the *gauche* conformer. Indeed, DMC ZPE evaluations return the same value starting from both *trans* and *gauche* geometries. A semiclassical estimate of the first excited state starting from the *gauche* conformer provides a reduced energy difference with respect to the energy gap between conformers found by electronic structure calculations. This is also at odds with harmonic estimates, which anticipate an increased gap. In our view, the “leak” effect and the reduced energy difference eventually explain experimental discrepancies in ethanol investigations and the difficulty to isolate the two conformers even at low temperatures.

We also notice that this result points to a striking resemblance with glycine as discussed in one of our previous works.^61^ We found that the 8 identified isomers of glycine reduced to 4 couples of conformers once zero-point energy and nuclear dynamics effects were taken into account. However, the impact of this finding was minor compared to the one for ethanol because the three main and experimentally investigated conformers of glycine were still energetically well separated. We think these results, and especially those presented for ethanol, provide new insight on the chemistry of small organic molecules, demonstrating the need to take nuclear quantum effects into account.

We employed the new potential to study the motions of the −CH_3_ and −OH rotors at the quantum mechanical level. DMC and DVR results are in very good agreement, and the computed DVR wave functions confirm the presence of the “leak” effect. Furthermore, the previously suggested geared motion of the rotors is confirmed by our calculations, and the 2-D model of the torsions based on cuts through the full-dimensional potential provides reasonable energy levels when compared to experiment.

Finally, as a perspective and as anticipated, we notice that semiclassical calculations are able to evaluate the energy of vibrationally excited states, and therefore, given the high fidelity of the PES, they will be employed, together with MULTIMODE calculations, in a future work for determining ethanol fundamental frequencies of vibration.

## Supporting Information Available

Plot comparing correction, Δ*V*_*CC*–*LL*_ and DFT energies, harmonic frequencies for *trans*–*gauche* isomerization saddle points, functional form of the 2-D CH_3_ and OH torsional potential, plot showing results of DVR calculation for CH_3_ torsional potential, plot of torsional potential of methyl rotor for TS1 and TS2, OH torsional potential for θ = 0 and θ = 60 degrees, comparison of ground state OH torsional wave function between DVR and DMC calculations, schematic of OH torsional path (PDF)
