# Supplementary material for: Quantum Calculations on a New CCSD(T) Machine-Learned Potential Energy Surface Reveal the Leaky Nature of Gas-Phase Trans and Gauche Ethanol Conformers
Source: J Chem Theory Comput. 2022 Aug 11;18(9):5527–38. doi: 10.1021/acs.jctc.2c00760 (PMC9476654; doi:10.1021/acs.jctc.2c00760)
Supplement: Supplementary file 1 — ct2c00760_si_001.pdf [file ct2c00760_si_001.pdf]

Supporting Information for: Quantum Calculations on a New CCSD(T) Machine-Learned Potential Energy Surface Reveal the Leaky Nature of Gas-Phase *Trans* and *Gauche* Ethanol Conformers

Apurba Nandi,[*,†] Riccardo Conte,[*,‡] Chen Qu,[¶] Paul L. Houston,[*,§] Qi Yu,[‖] and Joel M. Bowman[*,†]

†*Department of Chemistry and Cherry L. Emerson Center for Scientific Computation, Emory University, Atlanta, Georgia 30322, U.S.A.*

‡*Dipartimento di Chimica, Università Degli Studi di Milano, via Golgi 19, 20133 Milano, Italy*

¶*Independent Researcher, Toronto, Canada*

§*Department of Chemistry and Chemical Biology, Cornell University, Ithaca, New York 14853, U.S.A. and Department of Chemistry and Biochemistry, Georgia Institute of Technology, Atlanta, Georgia 30332, U.S.A*

‖*Department of Chemistry, Yale University, New Haven, Connecticut 06520, U.S.A.*

E-mail: apurba.nandi@emory.edu; riccardo.conte1@unimi.it; plh2@cornell.edu; jmbowma@emory.edu

Comparison of energies between different basis sets

Table S1: Single-point energies (cm^{-1}) of four stationary points at different level of theory relative to *trans* minima.

Geom.	CCSD(T)-F12a/aVDZ	CCSD(T)/aVQZ	CCSD(T)/aVQZ[a]
Gauche	42	45	45
TS1	389	383	383
TS2	438	424	423

[a] From Ref. 1.

Comparison between correction and DFT energies

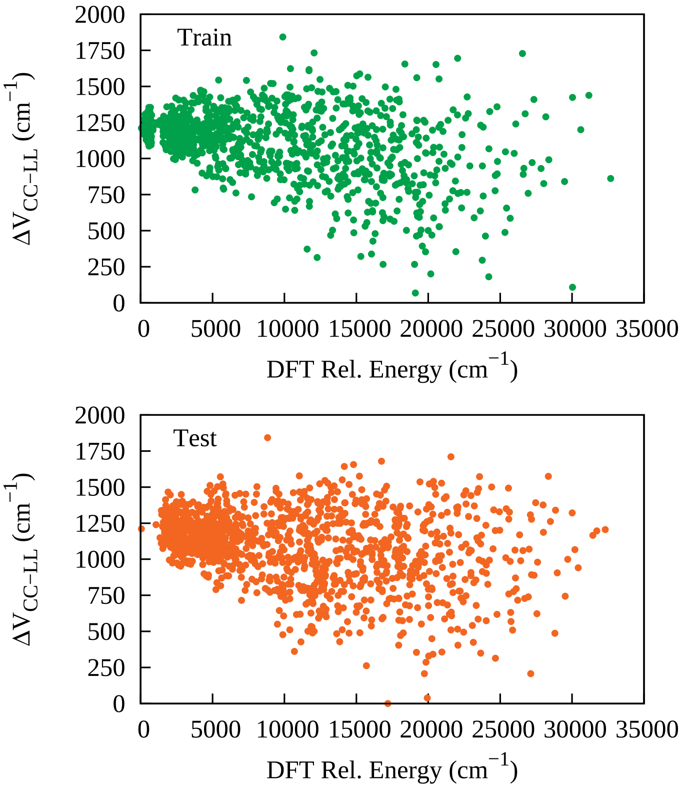

Figure S1: Plot of ΔV_{CC-LL} (relative to the reference value i.e. -35 732 cm^{-1}) vs DFT energy relative to the CH_3CH_2OH minimum value with the indicated number of training data sets.

Harmonic frequencies:

Isomerization TSs (Eclipsed and Syn)

Table S2: Comparison of harmonic frequencies (in cm^{-1}) between $V_{LL \to CC}$ PES and the corresponding *ab initio* (CCSD(T)-F12a/aug-cc-pVDZ) ones of both *eclipsed* and *syn* TSs of Ethanol.

	eclipsed			syn		
Mode	Δ-ML PES	*ab initio*	Diff.	Δ-ML PES	*ab initio*	Diff.
1	267i	287i	20i	332i	300i	-32i
2	261	256	-5	270	271	1
3	420	416	-4	411	414	3
4	800	797	-3	812	807	-5
5	899	899	0	892	892	0
6	1058	1064	6	1057	1061	4
7	1106	1106	0	1105	1109	4
8	1133	1132	-1	1186	1187	1
9	1285	1285	0	1307	1298	-9
10	1370	1358	-12	1308	1306	-2
11	1399	1397	-2	1406	1402	-4
12	1428	1427	-1	1446	1440	-6
13	1485	1486	1	1493	1493	0
14	1500	1598	-2	1507	1502	-5
15	1522	1520	-2	1534	1539	5
16	3020	3028	8	3015	3027	12
17	3028	3034	6	3027	3030	3
18	3059	3069	10	3054	3061	7
19	3112	3123	1	3103	3106	3
20	3123	3124	1	3109	3113	-6
21	3896	3890	-6	3872	3865	-7

Functional form for the 2-D CH_3 and OH torsional potential and calculations performed with it.

The functional form of the 2-D fit to the methyl and OH torsional motions shown in the 2-D contour plot of the main text is presented here. The best values of the variables in Table S3 were obtained by simultaneously fitting five cuts of the OH and CH_3 torsion calculated from the full-dimensional PES. There were two unknown parameters. These cuts are shown in Figs. 4 and 8 of the main text and in Fig. S2, below. The fits are virtually indistinguishable from the data and produced the values shown in the Table.

$$\begin{aligned}
V_{OH}(\phi) &= 0.5\sum_{n=1}^{4} V_{nOH}(1-\mathrm{Cos}(n\phi)), \\
V_{CH3}(\theta) &= V_{CH3}^{\phi=0}(0.5)(1-\mathrm{Cos}(3\theta)), \\
\mathrm{Correction}(\phi) &= 1+(\sum_{n=1}^{3} V_{nx}(1-\mathrm{Cos}(n\phi)) \\
V(\theta,\phi) &= V_{CH3}(\mathrm{Correction}(\phi))\times(0.5)(1-\mathrm{Cos}(3\theta))+V_{OH}(\phi)
\end{aligned} \tag{S1}$$

where the values of the constants are listed in the Table below.

Table S3: Constants for the two-dimensional potential for the OH and CH_3 torsion in ethanol.

Constant in Eq. (1)	Value (cm^{-1})
V_{1x}	0.0653
V_{2x}	0.000147
V_{3x}	0.00827
V_{CH3}	1208.4
V_{1OH}	86.3
V_{2OH}	-4.37
V_{3OH}	381.9
V_{4OH}	-32.7

The 1-D DVR results for the OH torsional potential have been shown in Fig. 8 of the

main text. As mentioned there, the only adjustable parameter is the moment of inertia for the rotor, which was taken to be $2.7/(N_{AV}m_e)$. A 1-D DVR result for the CH_3 potential is shown in Figure S3. The moment of inertia for the methyl rotor was taken here to be $10.5/(N_{AV}m_e)$.

Given the 2D potential in Eq. (S1) and the parameters in Table S3, we can predict how the OH torsion will vary as a function of the CH_3 torsional angle θ, as shown in Fig. S4. Not surprisingly, the barriers and the gauche conformation increase in energy as the methyl rotates so that one CH bond eclipses the OH bond. The figure demonstrates substantial interaction between the methyl and OH torsional motions.

Finally, we can also perform a 2-D DVR calculation[2] using the model 2-D potential. The previously described moments of inertia were adjusted to obtain the best fit. Results are shown in the Table in the main text.

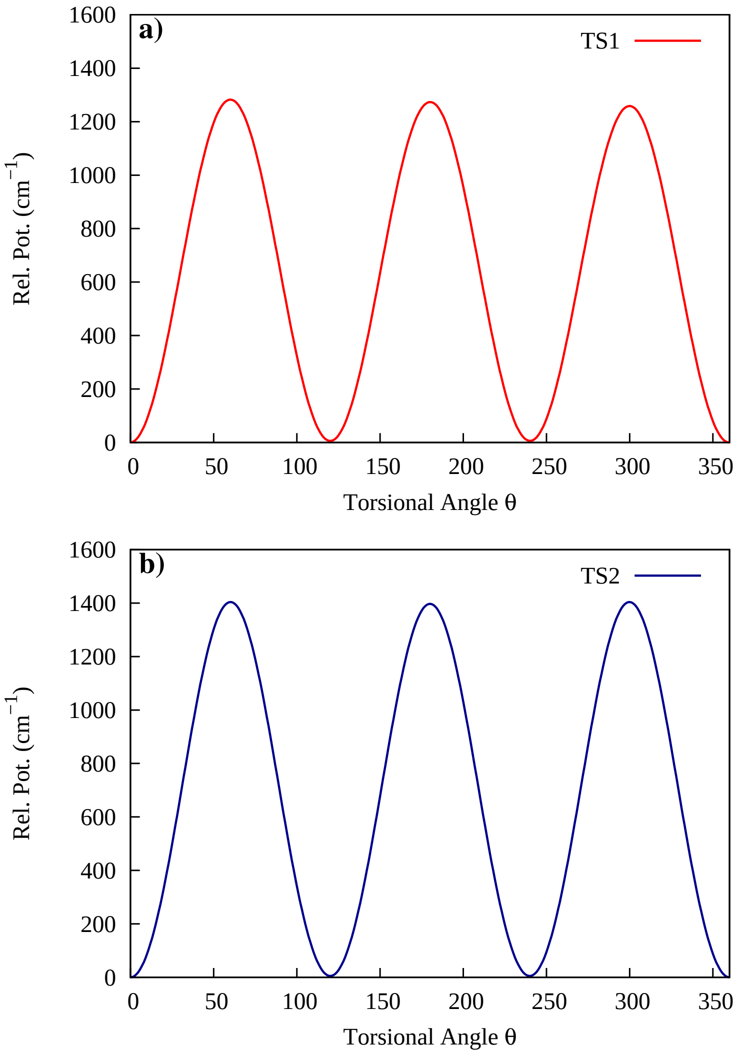

Figure S2: Torsional potential (not fully relaxed) of the methyl rotor of TS1 (a) and TS2 (b) geometry of Ethanol.

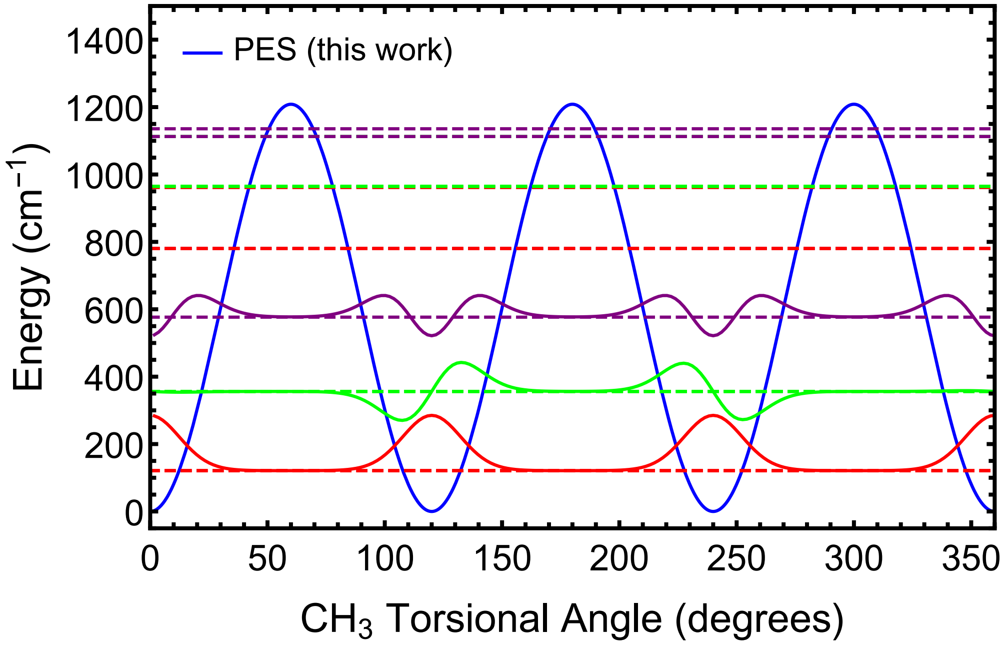

Figure S3: 1-D DVR results for the CH_3 torsional potential, whose potential is shown in the blue curve. The energy levels are shown as dotted lines, while the wavefunctions for the lowest three levels are shown as solid red, green, and purple lines.

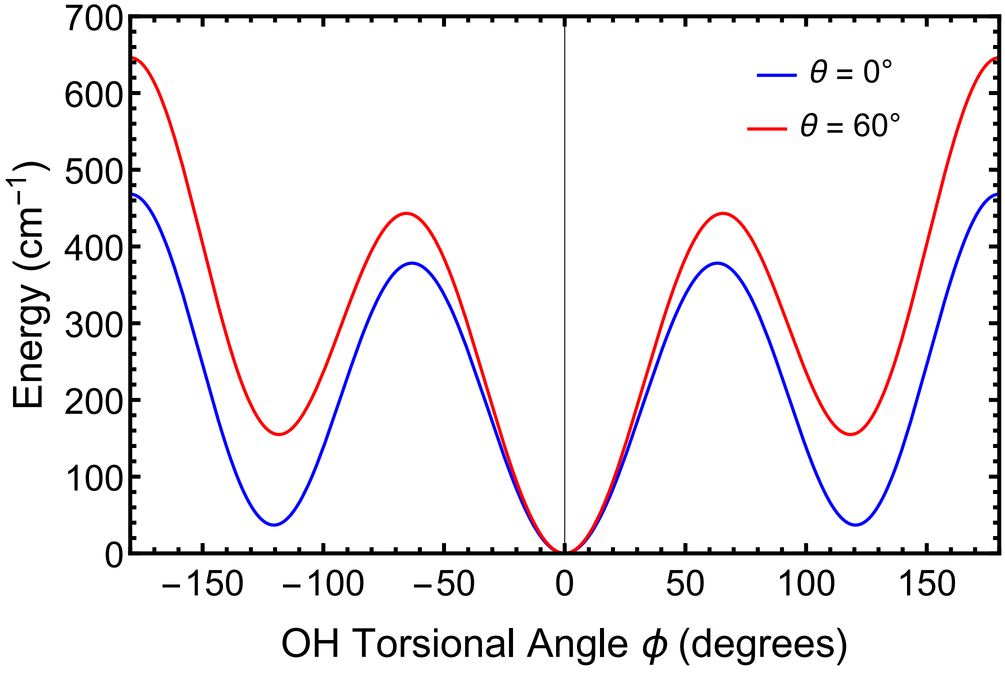

Figure S4: OH torsional potential for $\theta = 0$ and $\theta = 60$ degrees, normalized to have the same minimum.

Comparison of DVR and DMC torsional wavefunctions

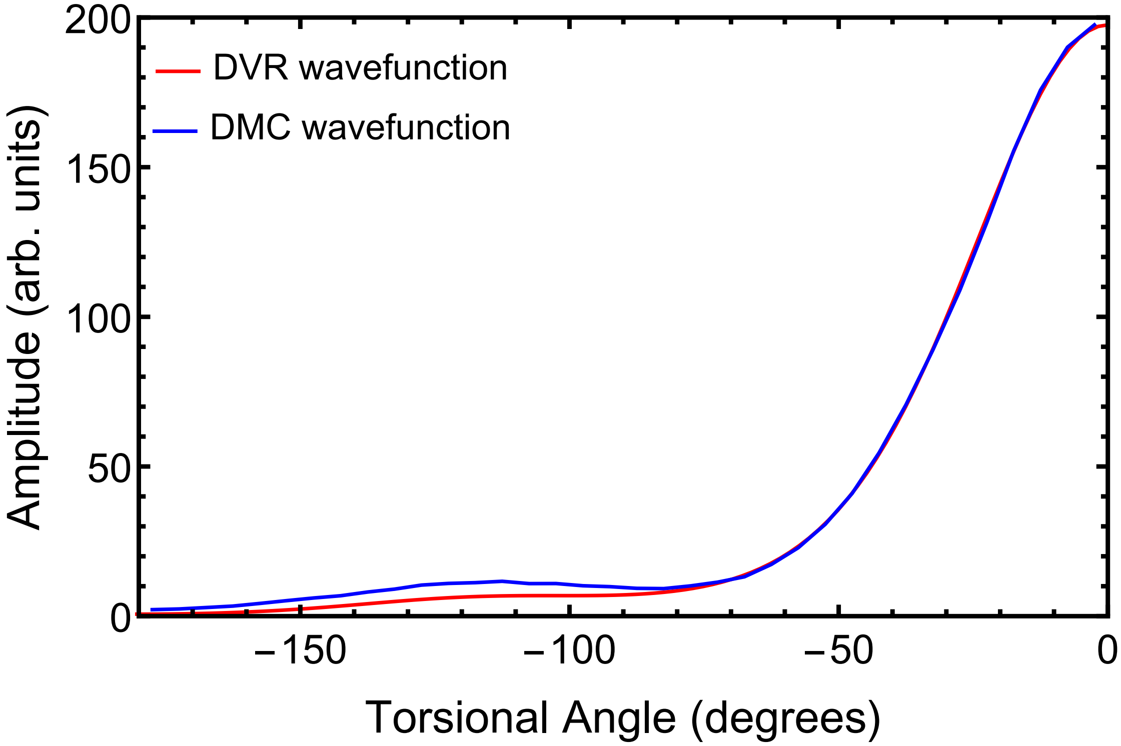

Figure S5: Comparison of the ground state OH torsional wavefunctions as determined from Discrete Variable Representation calculation on a 1-D cut (red) and from Diffusion Monte Carlo calculations on the full-dimensional PES (blue). Note that both wavefunctions have substantial amplitude near 120°, the geometry of the *gauche* state.

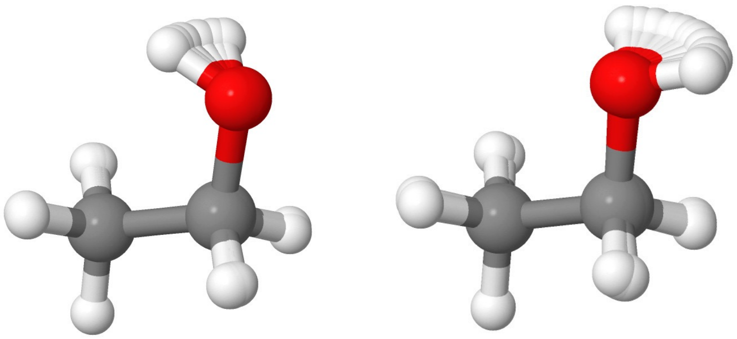

Figure S6: Snapshots of OH torsional path.

DFT vs CCSD(T) PES Comparison

Table S4: Comparison of energetics (in cm^{-1}) between DFT PES[3] (V_{LL}) and CCSD(T) PES (Δ-ML) relative to *trans* minimum energy.

Geom.	V_{LL}-PES[a]	Δ-ML PES	Diff.
Gauche	20	38	18
TS1	377	342	-35
TS2	472	491	21
$-CH_3$ Torsional Barrier	1180	1174	-6

[a] From Ref. 3.

Table S5: Comparison of harmonic frequencies (in cm^{-1}) between DFT PES[3] (V_{LL}) and CCSD(T) PES (Δ-ML) of both *trans* and *gauche* isomers of Ethanol.

	Trans			*Gauche*		
Mode	V_{LL}-PES[a]	Δ-ML PES	Diff.	V_{LL}-PES[a]	Δ-ML PES	Diff.
1	237	242	5	267	268	1
2	269	273	4	279	278	-1
3	417	417	0	422	425	3
4	820	817	-3	804	804	0
5	896	909	13	882	895	13
6	1035	1055	20	1057	1075	18
7	1094	1115	21	1075	1094	19
8	1176	1181	5	1133	1144	11
9	1266	1284	18	1280	1290	10
10	1299	1302	3	1368	1375	7
11	1402	1403	1	1403	1406	3
12	1446	1454	8	1416	1424	8
13	1483	1488	5	1487	1490	3
14	1498	1500	2	1494	1496	2
15	1524	1530	6	1515	1519	4
16	2978	2995	17	2989	3007	18
17	3005	3029	24	3015	3020	5
18	3031	3036	5	3068	3089	21
19	3098	3120	22	3087	3108	21
20	3105	3126	21	3100	3121	21
21	3843	3862	19	3826	3845	19

[a] From Ref. 3.

Table S6: Comparison of harmonic frequencies (in cm^{-1}) between DFT PES[3] (V_{LL}) and CCSD(T) PES (Δ-ML) of both *eclipsed* and *syn* TSs of Ethanol.

	Eclipse			*Syn*		
Mode	V_{LL}-PES[a]	Δ-ML PES	Diff.	V_{LL}-PES[a]	Δ-ML PES	Diff.
1	261i	267i	6i	336i	332i	-4i
2	259	261	2	270	270	0
3	420	420	0	408	411	3
4	801	800	-1	812	812	0
5	887	899	12	878	892	14
6	1036	1058	22	1044	1057	13
7	1087	1106	19	1079	1104	25
8	1126	1133	7	1182	1186	4
9	1275	1285	10	1292	1307	15
10	1360	1370	10	1306	1308	2
11	1397	1399	2	1405	1406	1
12	1423	1427	4	1440	1446	6
13	1481	1485	4	1491	1493	2
14	1498	1500	2	1502	1507	5
15	1517	1522	5	1531	1534	3
16	3003	3020	17	3010	3015	5
17	3023	3028	5	3013	3027	14
18	3035	3059	24	3034	3054	20
19	3091	3112	21	3082	3103	21
20	3102	3122	20	3088	3109	21
21	3875	3896	21	3853	3872	19

[a] From Ref. 3.

References

(1) Kirschner, K. N.; Heiden, W.; Reith, D. Small Alcohols Revisited: CCSD(T) Relative Potential Energies for the Minima, First- and Second-Order Saddle Points, and Torsion-Coupled Surfaces. *ACS Omega* **2018**, *3*, 419–432.

(2) Colbert, D. T.; Miller, W. H. A novel discrete variable representation for quantum mechanical reactive scattering via the S-matrix Kohn method. *J. Chem. Phys.* **1992**, *96*, 1982–1991.

(3) Houston, P. L.; Qu, C.; Nandi, A.; Conte, R.; Yu, Q.; Bowman, J. M. Permutationally invariant polynomial regression for energies and gradients, using reverse differentiation, achieves orders of magnitude speed-up with high precision compared to other machine learning methods. *J. Chem. Phys.* **2022**, *156*, 044120.
